# Prevalence and Factors Related to Trichomoniasis, Candidal Vaginitis, and Bacterial Vaginosis in Northeast Iran

**DOI:** 10.1155/ijm/9573665

**Published:** 2025-04-10

**Authors:** Mitra Salehi, Mohammadhassan Minooeianhaghighi, Alireza Mohammadzadeh, Azamsadat Mahmoudian, Simin Tayarani, Saeed Erfanpoor, Nasim Khajavian, Narjes Bahri, Morteza Rostamian

**Affiliations:** ^1^Vector-Borne Diseases Research Center, North Khorasan University of Medical Sciences, Bojnurd, Iran; ^2^Department of Microbiology, School of Medicine, Infectious Diseases Research Center, Gonabad University of Medical Sciences, Gonabad, Iran; ^3^Department of Obstetrics and Gynecology, School of Medicine, Bohlool Hospital, Gonabad University of Medical Sciences, Gonabad, Iran; ^4^Student Research Committee, Gonabad University of Medical Sciences, Gonabad, Iran; ^5^Department of Epidemiology, Faculty of Medicine, Infectious Diseases Research Center, Gonabad University of Medical Sciences, Gonabad, Iran; ^6^Department of Epidemiology and Biostatistics, School of Health, Social Determinants of Health Research Center, Gonabad University of Medical Sciences, Gonabad, Iran; ^7^Department of Midwifery, Faculty of Medicine, Social Determinants of Health Research Center, Gonabad University of Medical Sciences, Gonabad, Iran; ^8^English Department, Faculty of Medicine, Infectious Diseases Research Center, Gonabad University of Medical Sciences, Gonabad, Iran

**Keywords:** bacterial vaginitis, candidiasis, *Trichomonas*, vaginitis

## Abstract

*Candida* species, *Trichomonas vaginalis*, and bacteria are considered to be the main causes of vaginitis. This study investigated the prevalence of candidal, trichomonad, and bacterial vaginitis and factors related to infection in women. This cross-sectional study was conducted to investigate the relationship between different individual characteristics and common vaginal infections, namely, candidiasis, trichomoniasis, and bacterial vaginosis. The sample included all women referred to Allameh Bohlool Gonabadi Hospital women's clinic in Gonabad in 2021. After the patient's physical examination and questionnaire completion, samples were taken from the mucous secretions of the cervical vagina and the posterior fornix region using three sterile swabs. The first swab of secretions was placed on three glass slides for microscopic examination. At the same time, the second swab was transferred to the special *Trichomonas* culture medium (Dorset culture medium) available at the sampling site, observing sterile conditions. The third swab was placed in a test tube with a screw cap containing 5 cc of sterile physiological serum to be transferred to the laboratory. After adding two drops of potassium hydroxide (for elucidation), the first slide was examined under the microscope for the presence of *Candida* mycelia and buds. The second slide was used for warm staining to diagnose bacterial vaginosis. The third slide was used for Giemsa staining to detect *Trichomonas*. The swab in the screw-capped tube was stretched in Sabouraud Dextrose Agar (SDA) medium and kept in an incubator at 35° for 48 h to detect *Candida*. The prevalence of candidal, trichomonad, and bacterial vaginitis in the admitting women was 5%, 38.5%, and 5.8%, respectively. A significant relationship was found between the history of vaginal infection, trichomonad infection, and candidal infection (*p* = 0.03). Moreover, significant relationships were observed between bacterial infection and the husband's occupation (*p* = 0.002), methods of preventing pregnancy (*p* = 0.01), and menopause (*p* = 0.001). Vaginal infections are one of the common problems in women of all ages, and by knowing the factors that cause these infections, a big step can be taken to reduce the problem.

## 1. Introduction

Vaginal infection and vaginal discharge are one of the most common complaints that urge patients to visit women's and midwifery clinics. Attention and knowledge of the pathophysiology of these diseases and having an effective method for diagnosis can lead to the establishment of appropriate treatment and reduce complications and free the patient from their symptoms [1–4].

The most common infectious causes of vaginitis are bacterial agents, trichomoniasis, vaginosis, and vulvovaginal candidiasis [5]. In several studies between 1996 and 2003, bacterial vaginosis (22%–50%), vulvovaginal candidiasis (17%–39%), and trichomoniasis (4%–35%) were diagnosed in symptomatic women [6]. In Ziaei et al.'s study in Sari for sexually transmitted diseases, the prevalence of candidiasis infections was estimated at 46.6%, trichomoniasis at 7.3%, and bacterial vaginosis at 46.1% [7].

Bacterial vaginosis is the most common cause of abnormal vaginal secretions in women of reproductive age [8]. Fifty percent to 70% of patients complain about unpleasant vaginal odor (fishy or moldy), and increased vaginal discharge also occurs. The onset of bad odor and vaginal discharge in this infection has a uniform distribution throughout the menstrual cycle, and the discharge has a gray–white to yellowish–white color and sticks to the wall of the valve or vagina, and 50% of patients have no clinical symptoms [9]. Having a new sexual partner, smoking, using an intrauterine contraception (IUD), frequent alkalizing of the vagina due to frequent sexual intercourse or vaginal douching, continuous use of condoms, and exposure to chronic stress are among the risk factors for bacterial vaginosis [10–13]. The vaginal infection has been associated with serious health problems including severe pregnancy outcomes such as premature delivery and low birth weight babies, increased risk of pelvic inflammatory disease, and infection after abortion [14]. Women with bacterial vaginosis are more likely to suffer from herpes simplex virus 2 (HSV-2), *Trichomonas vaginalis*, *Neisseria gonorrhoeae*, and acquired immunodeficiency virus infections [8]. Vulvovaginal candidiasis is one of the most significant fungal diseases among women, which can even be transmitted from an infected woman to her sexual partner and cause painful sexual problems [15]. Seventy-five percent of women have experienced at least one episode of vulvovaginal candidiasis [16]. The symptoms of the disease include itching and burning of the valve and harmless thick white secretions [17]. Predisposing factors include diabetes, obesity, weakened immune system, frequent use of vaginal douches, use of broad-spectrum antibiotics, and stressful life [18] which create changes in the vaginal environment that accelerate the initiation of disease [19].

Another vaginal infection is trichomoniasis vaginosis, which is caused by the parasite *Trichomonas vaginalis*. Clinical symptoms include greenish–yellow frothy discharge, purulent discharge, urinary burning, and strawberry-shaped cervix [20, 21]. This infection is considered one of the causes of premature delivery and rupture of the amniotic sac and cervical and endometrial infections following surgery [22].

Although several studies have investigated the relationship between various individual factors and common vaginitis, the results were contradictory and inconclusive. In Valiani et al.'s study, factors such as occupation, level of education, body mass index, and weight had a significant relationship with vaginitis [23]. Conversely, Sehhatie-Shafaie and Namazi mentioned variables such as the number of births, the number of sexual intercourses, breastfeeding status, and pregnancy prevention methods as risk factors for candidiasis. Further, the age of marriage and sexual and personal hygiene were stated as risk factors for trichomoniasis [24]. In Balamurugan and Bendigeri's study on reproductive system infections in reproductive age, it was stated that birth rate, inappropriate social economic conditions, and poor menstrual hygiene have a direct effect on these infections [25]. To obtain an all-inclusive view [26–30], this study is aimed at investigating the prevalence of fungal, bacterial, and parasitic infections and their correlation with different demographic factors.

## 2. Methods

### 2.1. Study Design

This cross-sectional study was conducted in 2021, and the study protocol was approved by the Medical Ethics Committee of Gonabad University of Medical Sciences (IR.GMU.REC.1398.116).

### 2.2. Participants and Setting

Participants included married and nonpregnant women of different age groups and social statuses with complaints related to genital infections referred to Allameh Bohlool Gonabadi Hospital women's clinic in Gonabad. The inclusion criteria were informed consent; marriage history; absence of pregnancy; absence of vaginal bleeding; symptoms such as itching and burning in the genital area, increased secretions, bad smell, and color change; not using antibiotics, vaginal creams, or suppositories; and no vaginal douching within 48 h before taking the sample. The exclusion criteria were uncompleted questionnaires and unfavorable sample quality.

### 2.3. Sample Size and Sampling Technique

According to the study by Rezaei et al. [31], the sample size was calculated to be equal to 373 participants (using *n* = *Z*^2^ *P* *q*/*d*^2^, wherein *n* is the sample size, *P* is the estimated prevalence, *q* = 1 − *P*, *Z* = 1.96 for a confidence level (*α*) of 95%, and *d*^2^ is the margin of error (*N* = 1.96^2^ × 0.2 × 0.8/(0.04)^2^ = 373)). The total final sample size amounted to 400, taking into account 10% attrition, and purposive sampling was used.

### 2.4. Data Collection and Laboratory Procedure

The patient was placed in a lithotomy position for the examination, and after a physical examination using a speculum and observing whether the secretions were homogeneous or nonhomogeneous, samples were taken from the mucous secretions of the cervical vagina and the posterior fornix area using three sterile swabs. All vaginal sampling was performed by a gynecologist.

The first swab of secretions was placed on three glass slides for microscopic examination. At the same time, the second swab was transferred to the special *Trichomonas* culture medium (Dorset culture medium) available at the sampling site, observing sterile conditions. The third swab was placed in a test tube with a screw cap containing 5 cc of sterile physiological serum to be transferred to the laboratory. After transferring to the laboratory, the first slide was examined under a microscope after adding two drops of potassium hydroxide (for elucidation) in terms of the presence of *Candida* mycelia and buds. The second slide was used for Gram staining to diagnose bacterial vaginosis for detection of clue cells (epithelial cells covered by adherent Gram-negative bacilli). The third slide was used for Giemsa staining to detect *Trichomonas*. The swab in the screw-capped tube was stretched in Sabouraud Dextrose Agar (SDA) medium and kept in an incubator at 35° for 48 h to detect *Candida*. The Dorset culture medium was also incubated at 37° for 72 h to detect trichomoniasis.

The women were considered as having candidiasis if in addition to clinical symptoms, in direct observation of their sample or the culture medium, *Candida* was observed. They were considered positive cases of trichomoniasis when, in addition to clinical symptoms, the *Trichomonas* organism was observed in the microscopic observation of the slide prepared from the Dorset culture medium or in the Giemsa staining. Out of 20 samples that were positive for *Trichomonas vaginalis* diagnosis, parasites were seen in 17 samples (85%) but 20 positive samples were found in *Trichomonas vaginalis* parasite culture medium. Thus, as in previous studies, because the culture method for detecting *Trichomonas vaginalis* has a higher sensitivity, the culture method is preferred to the direct method for detecting the *Trichomonas vaginalis* parasite in cases where the samples detected negative in direct slide [32].

Bacterial vaginosis was diagnosed based on Amsel's criteria. The patients were considered positive if three of the four Amsel's criteria include homogeneous vaginal secretions, pH of vaginal secretions above 4.5, positive whiff test, and the presence of more than 20% of clue cells were detected.

### 2.5. Statistical Analysis

After collecting the data, SPSS Version 20 was used for statistical analysis. In the inferential analysis, chi-square was used to investigate the relationship between the prevalence of vaginitis in terms of qualitative variables at a significance level of less than 0.05.

## 3. Results

The mean age of the participants was 35.84 ± 8.90 years. The data revealed that out of 400 female patients with clinical symptoms, 135 patients (33.8%) were under 30 years old and 33 patients (8.3%) were over 49 years old; 229 patients (57.2%) lived in the city and 171 patients (42.8%) lived in the village. In terms of education level, 157 patients (39.2%) had a diploma, 142 patients (35.6%) had no diploma, and the rest had an education higher than a diploma. In terms of occupation, 292 patients (73%) were housewives and 108 patients (27%) were employed (Table 1).

In terms of infection with *Trichomonas vaginalis*, out of 400 women with clinical symptoms, 20 women (5%) were infected with this parasite by direct method and culture in a Dorset medium (Table 2).

Among 400 women with clinical symptoms, 154 (38.5%) were positive for candidal vaginitis and a significant relationship was observed between the history of vaginal infection and candidiasis infection (*p* = 0.03). Moreover, a significant relationship was observed between the history of vaginal infection and *Trichomonas* parasite infection (*p* = 0.03) (see Table 3). However, no significant relationship was found between *Trichomonas vaginalis* infection and age, place of residence, education level, job, husband's job, breastfeeding status, type of previous delivery, number of deliveries, number of sexual intercourses, length of the marriage, prevention method, menopause, diabetes, and antibiotic and corticosteroid use (*p* > 0.05). Similarly, no significant relationship was found between candidal infection and age, place of residence, education level, husband's job, breastfeeding status, previous birth, number of births, number of sexual intercourses and duration of the marriage, prevention method, menopause, diabetes, and antibiotic and corticosteroid use (*p* > 0.05).

Among the 400 women with clinical symptoms, 23 (5.8%) were positive for bacterial vaginosis. There was a significant difference between having bacterial vaginosis and age, place of residence, education level, job, breastfeeding status, history of vaginal infection, previous delivery, the number of deliveries, menopause (*p* = 0.001), husband's job (*p* = 0.002), and contraceptive method (*p* = 0.027).

## 4. Discussion

The results of this study showed that the prevalence of candidal, trichomonad, and bacterial vaginitis in the admitting women was 5%, 38.5%, and 5.8%, respectively, and a significant relationship was found between the history of vaginal infection, trichomonad infection, and candidal infection.

The findings showed that the prevalence of trichomoniasis infection was only 5%. The prevalence of trichomoniasis is high in developing and developed countries, especially among populations with high-risk behaviors, such as people with multiple sexual partners and prisoners [33]. The prevalence of this infection is 3.2% in Turkey, Libya, and Jordan. In Saudi Arabia, it reaches 28.1%, and in the United States, the prevalence of this infection among those visiting venereal disease clinics is about 25% [34–38]. In the studies conducted in Iran, the results revealed the prevalence of this infection in the following cities: Tabriz 2.9%, Tehran 3.2%, Babol 4%, Kashan 2%, Sari 1.1%, Shiraz 2.1%, Hamadan 1.2%, Karaj 1.1%, and Rasht 5.8% [24, 39–46]. The prevalence of trichomoniasis in our study was consistent with the results of previous studies, although it was slightly higher. The reason can be probably due to the difference in the time of conducting the studies, the difference in the studied population, the type of sample and methods used to detect the parasite, the difference in the level of health, and the cultural and social issues of these communities.

The results revealed that the prevalence of candidal vaginitis is 38.5%. In previous studies, the prevalence of candidal vaginitis was reported as follows: Shahrood 44%, Mashhad 43%, Torbet Jam 46.9%, Qom 35%, Talash 25%, and Karaj 11% [41, 47–51]. Since the prevalence of candidal vaginitis depends on health, cultural, and social factors, there is naturally a difference in the prevalence rate reported in different areas. Another speculation could be the difference in the method of examining samples and the number of examined samples. The high prevalence of this disease may be due to various reasons, including neglect of treatment and delay in going to the doctor and treatment measures, suitable backgrounds such as reproductive age and immune-decreasing diseases, and drug resistance in some candidal species [52].

The findings revealed the prevalence of bacterial vaginosis infection as 5.8%. The prevalence rate of bacterial vaginosis in Iran was reported as 18.9% [53]. The prevalence of this infection was reported as follows in different cities: Ardabil 12.4%, Shahrekord 6.8%, Shiraz 22.4%, Urmia 12.2%, Karaj 14.5%, Khalkhal 8%, and Kerman 37.7% [41, 54–58]. In preceding studies, the prevalence of bacterial vaginosis was also reported in different countries: India 31%, Japan 18.2%, Denmark 17%, Nigeria 17.3%, Kenya 37%, and Zambia 32.5% [53, 59–62].

The outcomes of this present study did not show a significant relationship between age and trichomoniasis (*p* = 0.81), vagino-bacterial infections (*p* = 0.17), and candidal vaginitis (*p* = 0.18). The findings are in line with Bakhtiaranjad et al. and Nazeri et al., and the prevalence of trichomoniasis was higher in women under 49 years and women aged 21–35 [41, 63]. Nevertheless, in other studies conducted in Iran, there was a significant relationship between age and trichomoniasis, and the prevalence of this infection was higher in the age group of 20–30 years than in others [39, 40]. In the studies conducted in France, America, and Japan, the prevalence of bacterial vaginosis in the younger age group was higher than in the other age groups [64, 65]. In Rahimi et al.'s study, the prevalence of bacterial vaginosis was higher at a young age (below 30 years) [54]. There was no correlation between age and bacterial vaginosis in the studies of Taghriri et al. and Golmohamadlou et al. [55, 57]. In some studies, similar to the present study, no significant relationship between age and candidal vaginitis has been seen [66–68], while in the study by Fati et al., the prevalence of candidal vaginitis (50%) was significantly higher in the age group of 25–35 years old [69].

The findings displayed no significant relationship between residence and trichomoniasis (*p* = 0.78), candidal vaginitis (*p* = 0.06), and bacterial vaginosis (*p* = 0.95). In line with these results, in other studies conducted in Iran, there was no correlation between these three types of vaginitis and place of residence [41, 63], which could be due to the expansion of wide-ranging health service centers and rural health centers and the promotion of prevention of these infections.

In this study, there was no significant relationship between education and trichomoniasis (*p* = 0.18), candidal vaginitis (*p* = 0.18), and bacterial vaginosis (*p* = 0.90) similar to some earlier studies [55, 57, 70]. However, in previous studies, low education level has been stated as one of the risk factors for trichomoniasis and bacterial vaginosis [31, 39, 41, 60, 63, 71] that underline the important role of health literacy in reducing infection. As a general indicator, literacy helps to prevent diseases and control all types of infections, which can be enhanced by more health awareness about sexually transmitted diseases.

The results also demonstrated that there is no significant relationship between a person's occupation and trichomoniasis (*p* = 0.17), candidal vaginitis (*p* = 0.47), and bacterial vaginosis (*p* = 0.68). However, the prevalence of these infections in housewives was higher than in others, which indicates the high level of awareness of working women and the impact of social factors on raising the level of women's knowledge. Similar to the results of this study, there was no significant relationship between occupation and vaginitis in preceding studies [31, 41, 63, 69, 72], though the results of other studies showed a significant relationship between a person's job and vaginal infections [31, 73–75].

In the present study, there was no relationship between the husband's occupation and trichomoniasis (*p* = 0.46) and candidal vaginitis (*p* = 0.50), which is in line with the results of Wang et al.'s study [76] which indicates there is no relationship between social and economic conditions with these infections. However, the results of the present study showed that there was a significant relationship between the husband's job and bacterial vaginosis (*p* = 0.020) and the prevalence of this infection in women whose husbands are self-employed (69.6%) and workers (26.1%) is more than in others. The results of other studies also showed a significant relationship between the husband's job and bacterial vaginosis [47, 72]. Considering the fact that the wife's job was an influential factor in bacterial vaginosis, it can be acknowledged that women with poor economic status have had less determination in treatment or performed treatments incompletely. This is due to various reasons such as lack of sufficient knowledge or access to health treatment information, lack of healthy nutrition, poor health condition environments, medical expenses, and lack of insurance.

In our study, there was no correlation between breastfeeding status and frequent vaginal infections (*p* > 0.05). The results of Shahinfar and Pour's study were consistent with our study, and no significant relationship between these two variables was reported (*p* > 0.05) [77].

The outcomes also disclosed a significant relationship between the history of vaginal infection and trichomoniasis (*p* = 0.03) and candidal vaginitis (*p* = 0.03). Thus, the incidence of these infections was higher in people who had a history of previous vaginal infections (*p* < 0.05). Moreover, the present study exhibited no significant relationship between the history of vaginal infection and bacterial vaginosis (*p* = 0.88). In Shahinfar and Pour's study, the results displayed no significant relationship between the prevalence of common vaginal infections and the history of vaginal infections [77]. Prevalence of vaginal infections in people who had a history of previous infections can be due to nonobservance of health tips or their lifestyle, as well as the readiness of the vaginal environment for the growth of microorganisms.

According to the findings, there was no significant relationship between the type of delivery and trichomoniasis (*p* = 0.62), candidal vaginitis (*p* = 0.36), and bacterial vaginosis (*p* > 0.36); nonetheless, the prevalence of vaginal infections in women with a history of natural childbirth was more than others. The results also indicated no significant relationship between the number of deliveries and vaginal infections (*p* > 0.05). In the same line, Tafazoli et al. found no correlation between the number of deliveries and bacterial vaginosis (*p* > 0.05) [72]. Nevertheless, Fatty et al. found a direct relationship between the number of births and candidal vaginitis (*p* < 0.05), and their results presented that during pregnancy, the conditions of the vagina are more open to the growth of *Candida* fungus [69].

The results of Golmohammadlou et al.'s study were consistent with our study and showed that the level of sexual activity was not related to the prevalence of bacterial vaginosis [57]. However, in other studies, the results showed a higher prevalence of bacterial vaginosis in women with more sexual activity than others [62, 72, 78–80] which indicates that a high pH of semen may increase the growth of *Gardnerella vaginalis* bacteria and increase the risk of bacterial vaginosis or its recurrence. Similarly, other possible causes of the recurrence of bacterial vaginosis may be bacterial sources inside the body, because sexual intimacy may facilitate the entry of microorganisms into the vagina [81].

The findings indicated that there was no significant relationship between the length of marriage and trichomoniasis (*p* = 0.62), candidal vaginitis (*p* = 0.36), and bacterial vaginosis (*p* > 0.36). In former studies, there was no significant relationship between the duration of the marriage and contracting these infections, and the prevalence of vaginal infections was higher in young couples with a history of marriage less than 5 years [17, 77].

The findings exposed that there was no correlation between the contraceptive method and trichomoniasis (*p* = 0.55) and candidal vaginitis (*p* = 0.38). However, the prevalence of these infections in people who used the natural method or did not use a special method was higher than in others. Other studies showed that the highest prevalence of candidal vaginitis was in women who used IUD or contraceptive pills, and the lowest prevalence of infection was in those who used Norplant or condoms [41, 51, 66, 82]. The results of Bakhtiari et al.'s study showed a lower prevalence of trichomoniasis in people who used condoms [40]. In the present study, a significant relationship was observed between bacterial vaginosis and the contraceptive methods (*p* = 0.027). In prior studies, the prevalence of bacterial vaginosis in people who used condoms as a contraceptive was significantly lower, and the IUD was an aggravating factor for vaginal discharge and infection [57, 72].

In our study, although there was no significant relationship between menopause and trichomoniasis (*p* = 0.43) and candidal vaginitis (*p* = 0.54), there was a significant relationship between bacterial vaginosis and menopause (*p* = 0.001). Besides, the prevalence of vaginal infections in postmenopausal women was lower than that in reproductive age. The results of Namazi et al.'s study showed that vaginal infections usually occur during menstruation, because the pH of the vagina is at a level that allows the colonization of organisms, but these conditions are not available during menopause [24].

With regard to diabetics, there was no significant relationship between diabetes and vaginal infections (*p* > 0.05). The results of preceding studies also showed no significant relationship between diabetes and vaginal infections. However, in these studies, diabetes has been stated as an underlying factor of candidal vulvovaginitis [49, 69, 83]. Diabetes is one of the predisposing factors of vaginal fungal disease. The lack of a relationship between diabetes and candidiasis in our study was probably due to the good control of diabetes in patients or the small sample size of people with diabetes.

Finally, the findings indicated that there was no significant relationship between the use of antibiotics, corticosteroids, and cytotoxic agents and the occurrence of vaginal infections, as well as between the use of tobacco and the occurrence of vaginal infections (*p* > 0.05), which were consistent with the results of former studies [54, 69, 77].

The findings imply that increasing people's awareness of the risks and consequences of vaginitis, the ways of its transmission, and the prevention of sexually transmitted diseases can play an important role in reducing the costs of public health treatment. One of the limitations of this study was the noncooperation of some research units for sampling.

## 5. Conclusions

In this study, a significant relationship was found between the history of vaginal infection, trichomonad infection, and candidal infection. Additionally, a significant relationship was observed between the husband's occupation, methods of preventing pregnancy, and menopause with a bacterial infection. Knowing the effective factors in generating vaginal infections can be an important step in reducing these infections.

## Figures and Tables

**Table 1:** Participants' demographic information (*N* = 400).

**Variable name**	**Variable levels**	**Frequency**	**Percent**
Age	Under 30 years	135	33.8
	30–39 years old	131	32.6
	40–49 years old	101	25.3
	More than 49 years	33	8.3

Residence	Town	229	57.2
	Village	171	42.8

Education	Less than a diploma	142	35.6
	Diploma	157	39.2
	Higher than a diploma	101	25.2

Job	Housewife	292	73
	Employed	108	27

Husband's job	Employed	395	98.75
	Unemployed	5	1.25

Breastfeeding status	Yes	14	3.5
	No	386	96.5

Tobacco smoking	Yes	17	4.3
	No	383	95.8

History of vaginal infection	Yes	249	62.3
	No	151	37.7

Birth control methods	Natural methods	186	46.5
	IUD, tablet, condom, or tubectomy	133	33.3
	None	81	20.3

Method of delivery	Natural	210	52.5
	Cesarean operation	118	29.5
	None	72	18

Number of delivery	One	122	30.5
	Two	123	30.8
	Three and more	83	21
	None	72	17.7

Number of intercourses	Once a week	129	32.2
	More than once a week	197	49.3
	Once every 2 weeks or less	74	18.5

Menopause	Yes	11	2.7
	No	389	97.3

Duration of marriage	Less than 11 years	234	58.5
	11 years and more	166	41.5

Diabetes	Yes	5	1.25
	No	395	98.75

Taking antibiotics and corticosteroids	Yes	14	3.50
	No	386	96.5

**Table 2:** Infections with trichomoniasis, candidal vaginitis, and bacterial vaginosis (*N* = 400).

**Infection**	**Number of infected**	**Percent**
Trichomoniasis	20	5
Candidal vaginitis	154	38.5
Bacterial vaginosis	23	5.8

**Table 3:** Correlations between types of infection and history of vaginal infection, husband's job, menopause, and birth control methods (*N* = 400).

**Types of infection**	**Variable**			**p** ** value**
Trichomoniasis (*N* = 20) (5)	History of vaginal infection			0.03
	Yes	No		
	17 (85)	3 (15)		

Candidal vaginitis (*N* = 154) (38.5)	History of vaginal infection			0.03
	Yes	No		
	106 (68.8)	48 (31.2)		

Bacterial vaginosis (*N* = 23) (5.8)	Husband's job			0.002
	Employed	Unemployed		
	23 (100)	0 (0)		
	Menopause			0.001
	Yes	No		
	4 (17.4)	19 (82.6)		
	Birth control methods			0.027
	Natural	Tablet, condom, IUD, or tubectomy	None	
	16 (69.6)	2 (8.7)	5 (21.7)	

## Data Availability

The datasets used during the current study are available from the corresponding author on reasonable request.
